# MED12 mutation as a potential predictive biomarker for immune checkpoint inhibitors in pan-cancer

**DOI:** 10.1186/s40001-022-00856-z

**Published:** 2022-10-29

**Authors:** Yong Zhou, Yuan Tan, Qin Zhang, Qianqian Duan, Jun Chen

**Affiliations:** 1 Department of Cardiothoracic Surgery, Nanjing Drum Tower Hospital, Medical School of Nanjing University, Nanjing, China; 2 The Medical Department, The State Key Lab of Translational Medicine and Innovative Drug Development, Jiangsu Simcere Diagnostics Co., Ltd, Nanjing Simcere Medical Laboratory Science Co., Ltd, Jiangsu Simcere Diagnostics Co., Ltd, Building 5, No. 699-18 Xuanwu Avenue, Xuanwu District, Nanjing, Jiangsu China; 3 The 1st Dept of Thoracic Medical Oncology, The Second Hospital Of Dalian Medical University, No. 467, Zhongshan Road, Shahekou District, Dalian, 116027 Liaoning China

**Keywords:** MED12, Biomarker, Immune checkpoint inhibitors, Pan-cancer, Anti-tumor immunity

## Abstract

Immune checkpoint inhibitors (ICIs) therapy elicits admirable anti-tumor responses across many types of cancer. Growing evidence point to a link to Mediator complex subunit 12 (MED12) and DNA damage repair (DDR) and TGF-β signing, while the clinical data on the association of MED12 and ICIs response are lacking. In this study, clinical and whole-exome sequencing (WES) data from published studies were merged as a WES cohort to explore the association between MED12 mutation (MED12-Mut) and ICIs efficiency across cancers. Then, Memorial Sloan Kettering Cancer Center (MSKCC) cohort was used for validating our findings. The Cancer Genome Atlas (TCGA) cohort was used to perform anti-tumor immunity and prognosis analysis. In the WES cohort (*n* = 474), significant differences were detected between MED12-Mut and MED12-wildtype (MED12-Wt) patients regarding durable clinical benefit (DCB, 80.00% vs. 53.67%, *P* = 0.022). In addition, significantly prolonged PFS was observed in MED12-Mut patients (mPFS: not reached, NR vs. 5.87 months, HR: 0.38, 95% CI 0.17–0.85, log-rank *P* = 0.015), After taking into account age, gender, metastasis, treatment and TMB status, the result of multivariable Cox proportional hazards regression showed significantly better PFS (HR:0.40, 95% CI 0.18–0.92; *P* = 0.031). In the MSKCC cohort (*n* = 1513), overall survival advantage was achieved in MED12-Mut patients (mOS: 41 vs. 19 months, HR:0.54, 95%CI 0.34–0.85; log-rank *P* = 0.007), after taking into account same factors in WES cohort, this link still existed (HR: 0.60, 95% CI: 0.38–0.96, *P* = 0.033), Notably, TMB was also found significantly higher in MED12-Mut patients in both WES and MSKCC cohort. Further tumor-infiltrating lymphocytes and DDR-related gene analysis revealed anti-tumor immunity in MED12-Mut patients. Totally, MED12-Mut successfully predicted better clinical outcomes in ICIs-treated pan-cancer cohort, indicating that MED12-Mut could serve as a potential predictive biomarker for immune checkpoint inhibitors in pan-cancer.

**Supplementary Information:**

The online version contains supplementary material available at 10.1186/s40001-022-00856-z.

To the editors,

Immune checkpoint inhibitors (ICIs) can elicit impressive improvement of survival in different cancer types. However, few patients can respond well to immunotherapy and reliable biomarkers are urgently needed [1]. Emerging evidence indicates mediator complex subunit 12 (MED12) together with the mediator complex, activates gene transcription in Wnt/β catenin signaling, which regulates anti-tumor immunity [2, 3]. However, clinical data on the association of alterations in transcriptional regulation-related genes and ICIs benefit are still lacking. Previous preclinical studies have shown that mediator complex subunit 12 (MED12), a component of the mediator transcription regulation complex, is associated with DNA damage repair (DDR) and TGF‑β receptor signaling [4, 5]. However, the role of MED12 in tumor immunotherapy is still uncertain. In this study, multidimensional data were used to probe the relationship between the efficacy of immunotherapy across cancers and MED12 mutations.

In the TCGA pan-cancer cohort, the average mutation frequency of MED12 was 3.35% and the specific mutation distribution in different cancers is shown in Additional file 2: Fig. S2. Then, to investigate whether the somatic mutations in MED12 were related to the response to ICIs, we collected the whole-exome sequencing (WES) data of 474 patients across 5 cancer types from 6 immunotherapy studies (http://www.cbioportal.org/) as a WES cohort (Additional file 1). The study flowchart, inclusion and exclusion criteria are shown in Additional file 1: Fig. S1. Clinical information of patients are shown in Additional file 5: Table S1, and the details of material and method was shown in Additional file 4. The results of assessment of the WES cohort revealed that MED12 mutant (MED12-Mut) patients had a significantly longer progression-free survival (PFS) (mPFS: not reached, NR vs. 5.87 months, HR: 0.38, 95% CI 0.17–0.85, log-rank *P* = 0.015, Fig. 1A) than that of MED12 wildtype (MED12-Wt) patients. This link still existed in the multivariate-adjusted Cox model incorporating age, gender, TMB level, cancer type, and treatment (HR: 0.40, 95% CI 0.18–0.92, *P* = 0.031; Fig. 1B). In addition, MED12-Mut patients had a better clinical response (ORR: 50.0% vs. 29.17%, *P* = 0.077; DCR: 80.0% vs. 53.67%, *P* = 0.022, Fig. 1C, D) than that of MED12-Wt patients. To validate the predictive value of MED12, the MSKCC cohort (*n* = 1513) was surveyed to determine the relationship between MED12-Mut and overall survival (OS). In the MSKCC cohort, MED12-Mut patients achieved significantly longer OS (mOS: 41 vs. 19 months, HR: 0.54, 95% CI 0.34–0.85, log-rank *P* = 0.007, Fig. 1E) than that of MED12-Wt patients. Even taking into account the same factors as the WES cohort, the multivariate-adjusted Cox model also demonstrated that MED12-Mut was associated with significantly better OS (HR: 0.60, 95% CI 0.38–0.96, *P* = 0.033; Fig. 1F). To explore the prognostic value of MED12, survival analysis was performed in the TCGA cohort (non-ICI treatment cohort) according to MED12-Mut status. No significant difference was found in OS between the ME12-Mut and MED12-Wt subtypes (mOS: 84.7 vs. 72.1 months, HR: 0.86, 95% CI 0.71–1.04; log-rank *P* = 0.12, Additional file 3: Fig. S3).

**Fig. 1:**
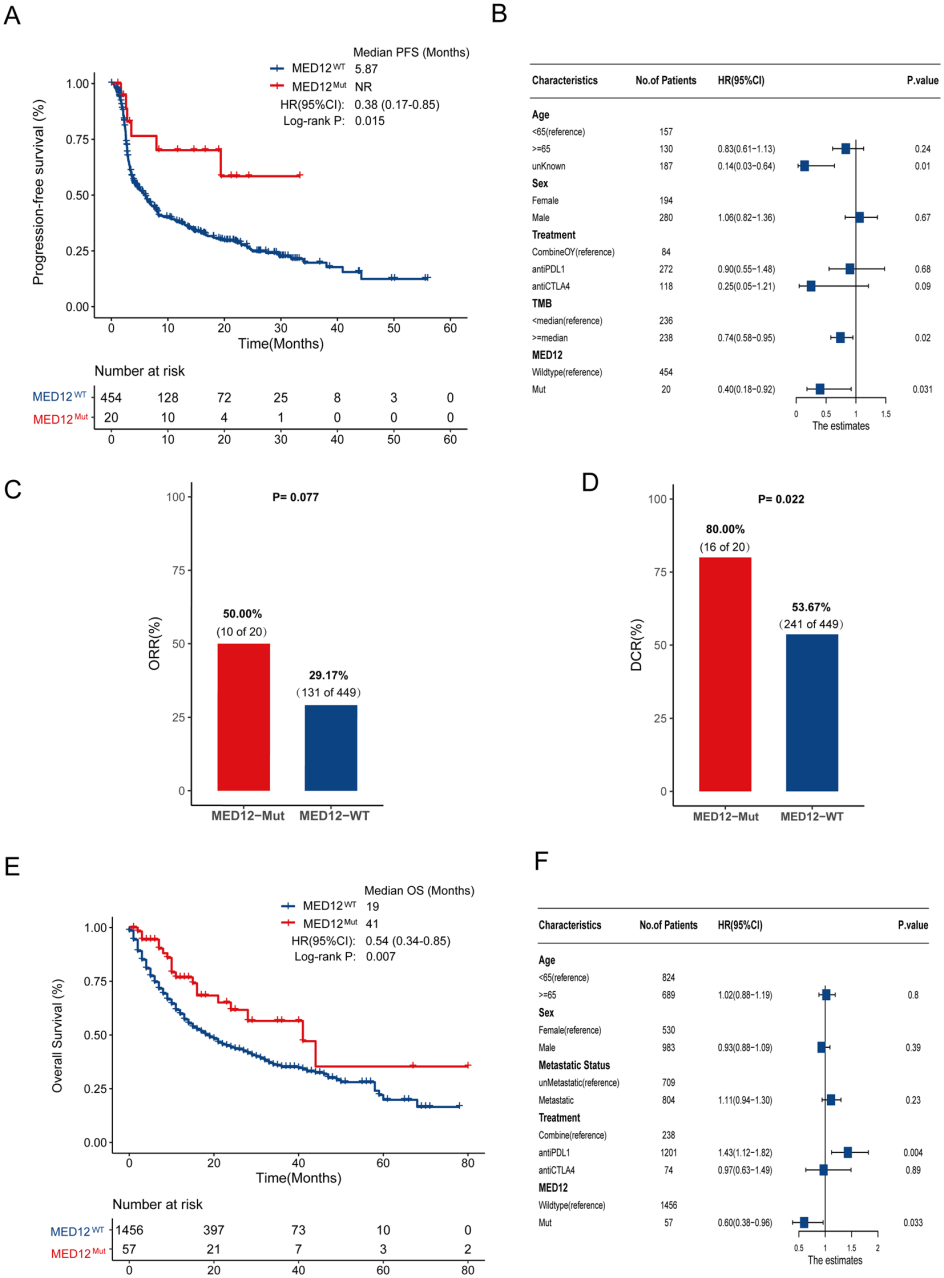
The predictive value of MED12 mutation in immunotherapy of pan-cancer. **A** Kaplan–Meier survival analysis comparing PFS between MED12-Mut and MED12-Wt patients in combination with the five WES cohorts. **B** Multivariate Cox regression analysis of MED12 mutations in WES cohort, the confounding factors including sex, age, metastasis status, treatment, TMB level, and cancer type were adjusted. The proportional hazards assumption was tested before the Cox regression used the Stratified Cox model to resolve independent variables that do not conform to the PH assumption. **C** Comparison of the ORR between the MED12-Mut and MED12-Wt groups from the WES cohorts. **D** Comparison of the DCR between the MED12-Mut and MED12-Wt groups from the WES cohorts. **E** Kaplan–Meier survival analysis comparing OS between MED12-Mut and MED12-Wt patients in the MSKCC cohort. **F** Multivariate Cox regression analysis of MED12 mutations in the combination of MSKCC cohort with age, sex, metastatic status, treatment type, TMB, and cancer types were taken into account. The proportional hazards assumption was tested before the Cox regression used the Stratified Cox model to resolve independent variables that do not conform to the PH assumption

Regarding the outstanding predictive value of MED12 in pan-cancer immunotherapy, the potential mechanisms were investigated. Tumor mutation burden (TMB) and tumor neoantigen burden (TNB), which are relevant tools for the identification of patients likely to respond to ICIs, were studied first. In the immunotherapy cohort and TCGA cohort, MED12-Wt tumors had a higher TMB than MED12-Wt tumors (all *P* < 0.05, Fig. 2A–C). In addition, in the WES cohort, patients with MED12-Mut harbored higher TNB (*P* = 0.007, Fig. 2D). A previous study showed that the loss of MED12 leads to a significant upregulation of the immune genes associated with DNA repair deficiency [4]. Therefore, the DDR gene set was used to explore the differences in DDR pathway variants between MED12-Mut and MED12-Wt samples. As expected, MED12-Mt samples had significantly more mutations in the DNA damage response (DDR) pathway (Fig. 2E). As a major component of the TME, immune infiltrates have been proven to contribute to tumor progression and immunotherapy responses [6]. Therefore, to further investigate the association between anti-tumor immunity and MED12-Mut across multiple cancer types, the link between MED12 mutation and infiltration of immune cells was explored. Tumor-infiltrating lymphocytes, especially CD8 T cells and dendritic cells (DC), were generally more abundant in MED12-Mut tumors than in MED12-Wt tumors across cancer types in TCGA (Fig. 2F). In fact, a large number of studies have indicated that the density of TILs is positively related to the immune response in various kinds of tumors. Increased infiltration of CD8 + T cells in tumors correlates with better outcomes [7, 8]. Dendritic cells are often associated with superior cross-presentation of antigens, which results in stronger CD8 + T cell immunity [9]. Our results showed that MED12 mutated patients had higher levels of CD8 T cells and dendritic cell infiltration, which also indicated the increased tumor immunogenicity in MED12 mutated patients.

**Fig. 2:**
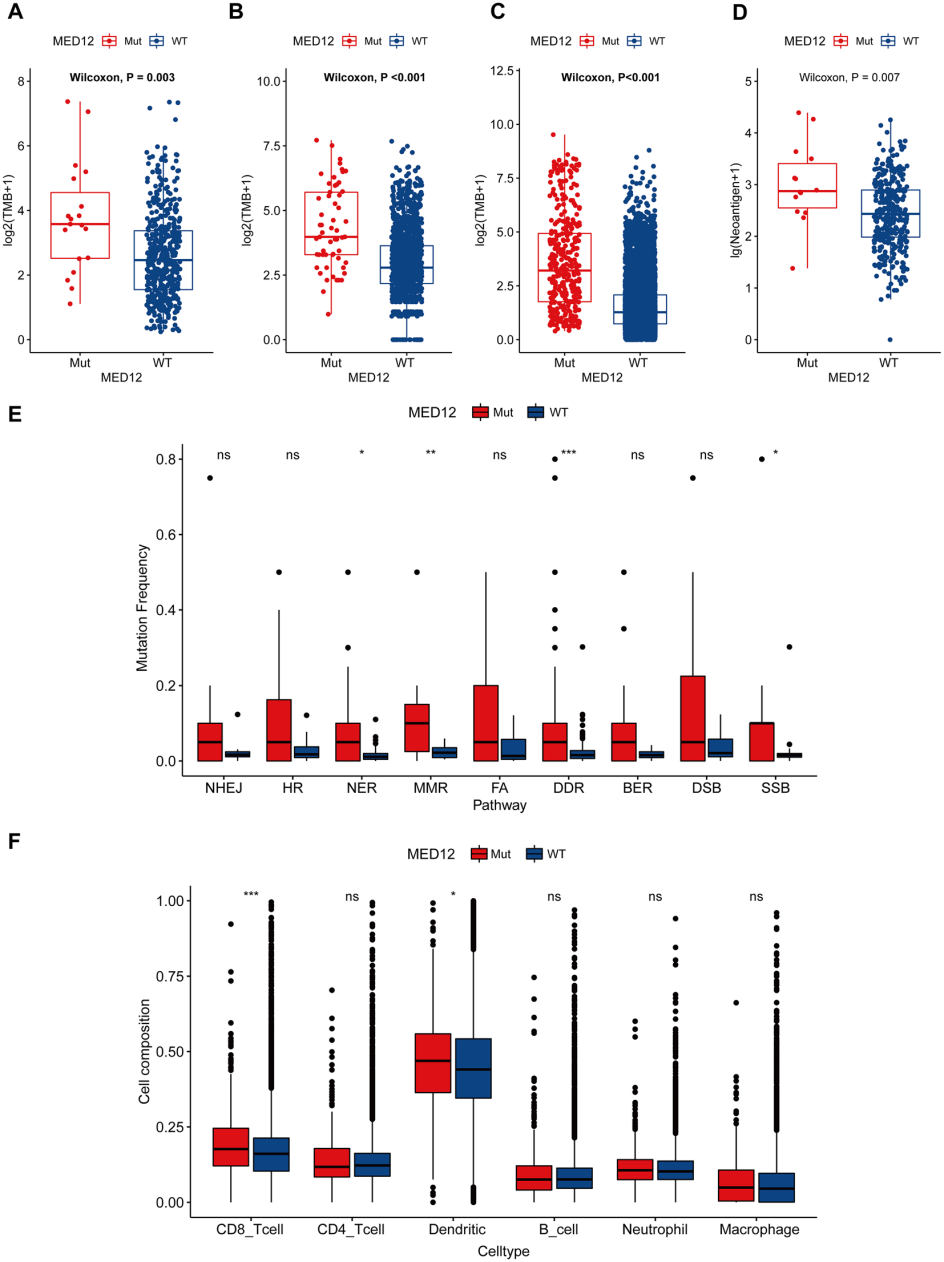
Relationship between MED12 mutation and enhanced tumor immunity. **A** Comparison of the TMB between the MED12-Mut and MED12-Wt groups from the WES cohorts. **B** Comparison of the TMB between the MED12-Mut and MED12-Wt groups from the MSKCC cohort. **C** Comparison of the TMB between the MED12-Mut and MED12-Wt groups from the TCGA cohort. **D** Comparison of the TNB between the MED12 mutant and wildtype groups from the WES cohorts. **E** Comparison of DNA damage-related gene (DDR) set variants between MED12-Mut and MED12-Wt patients in TCGA cohort. **F** Comparison of tumor-infiltrating immune cells abundance in MED12-Mut and MED12-Wt pan-cancers. *P < 0.05; **P < 0.01; ***P < 0.001; ns: *P* > 0.05

In summary, our study provides solid evidence that MED12 mutation is associated with a better clinical outcome of pan-cancer immunotherapy. Therefore, MED12 mutation has the potential to serve as a predictive biomarker for immune checkpoint inhibitors across cancers. Validation of the predictive value in future prospective trials and exploration of the molecular mechanism in further molecular research are warranted for MED12.

## Supplementary Information

**Additional file 1: Fig. S1.** Flowchart of the study design. A. Merge of WES cohorts from five published studies (Hellman et al. [10], Rizvi et al. [11], Miao et al [12, 13], Allen et al. [14], Liu et al. [15]). B. MSKCC cohort from the published study (Samstein et al [16]). C. The TCGA dataset was used to perform DDR-related gene mutation, tumor-infiltrating immune cells and prognostic analyses.

**Additional file 2: Fig. S2.** The pan-cancer landscape of MED12 mutations across human tumors. The proportion of MED12 mutated tumors identified for each cancer type with alteration frequency in TCGA pan-cancer cohorts.

**Additional file 3: Fig. S3. **Kaplan–Meier curves of OS between the MED12-Mut and wildtype groups in the TCGA cohort.

**Additional file 4:** Materials and methods

**Additional file 5: Table S1.** Detailed clinical information of the five WES cohorts and the MSKCC cohort.

## Data Availability

The materials of patient cohorts used for the current study were publicly available and can be accessed by the TCGA and CbioPortal database (https://portal.gdc.cancer.gov/, https://www.cbioportal.org/).

## Abbreviations

ICIs: Immune checkpoint inhibitors
MED12: Mediator complex subunit 12
MED12-Mut: MED12 mutation
MED12-Wt: MED12 wildtype
DDR: DNA damage repair
WES: Whole-exome sequencing
TCGA: The Cancer Genome Atlas
MSKCC: Memorial Sloan Kettering Cancer Center
PFS: Progression-free survival
OS: Overall survival
TMB: Tumor mutation burden
