# Supplementary figures and images for: MED12 mutation as a potential predictive biomarker for immune checkpoint inhibitors in pan-cancer
Source: Eur J Med Res. 2022 Oct 29;27:225. doi: 10.1186/s40001-022-00856-z (PMC9618186; doi:10.1186/s40001-022-00856-z)

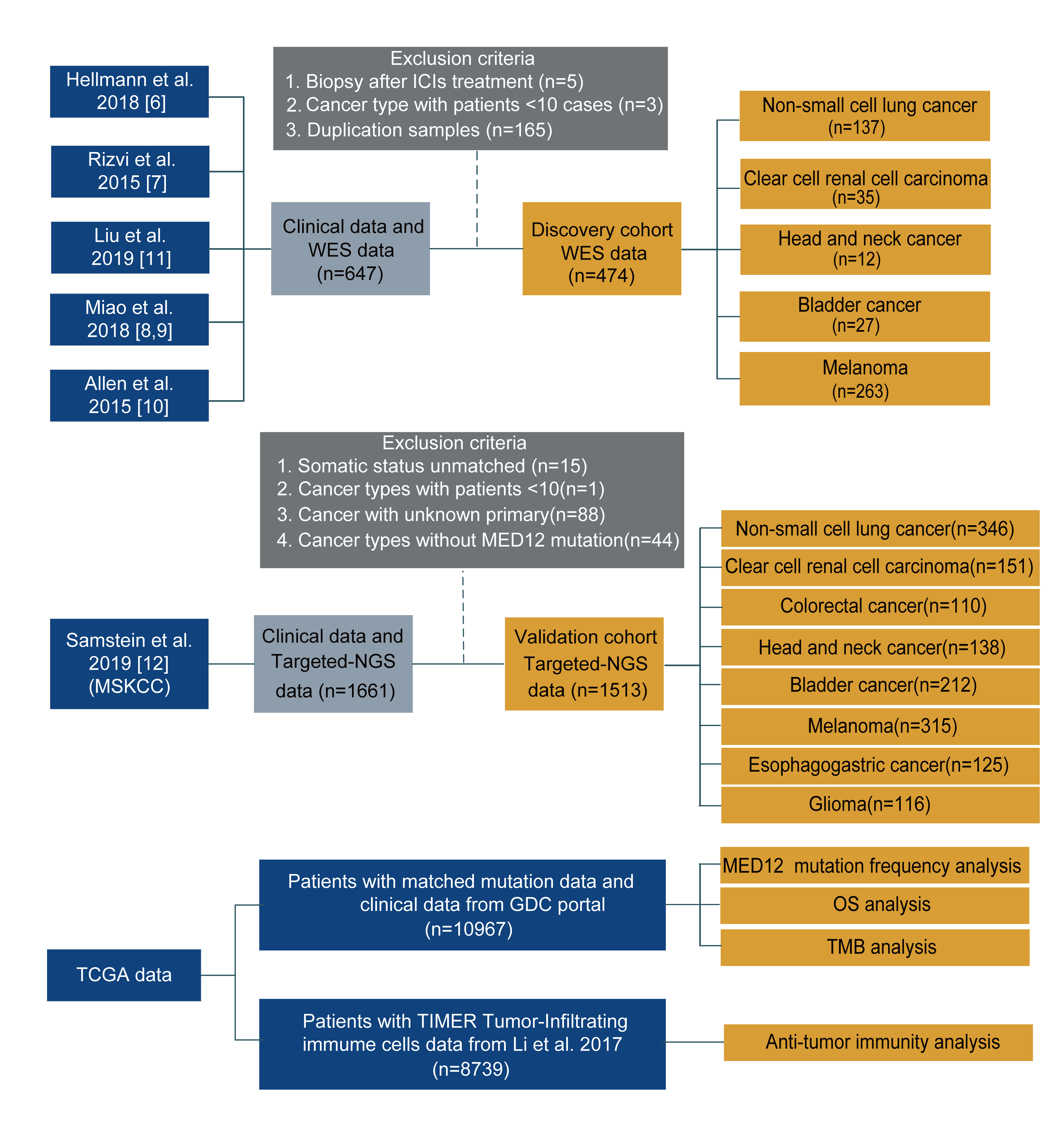

Supplement: Supplementary file 1 — Additional file 1: Fig. S1. Flowchart of the study design. A. Merge of WES cohorts from five published studies (Hellman et al. [10], Rizvi et al. [11], Miao et al [12, 13], Allen et al. [14], Liu et al. [15]). B. MSKCC cohort from the published study (Samstein et al [16]). C. The TCGA dataset was used to perform DDR-related gene mutation, tumor-infiltrating immune cells and prognostic analyses. [file 40001_2022_856_MOESM1_ESM.png]

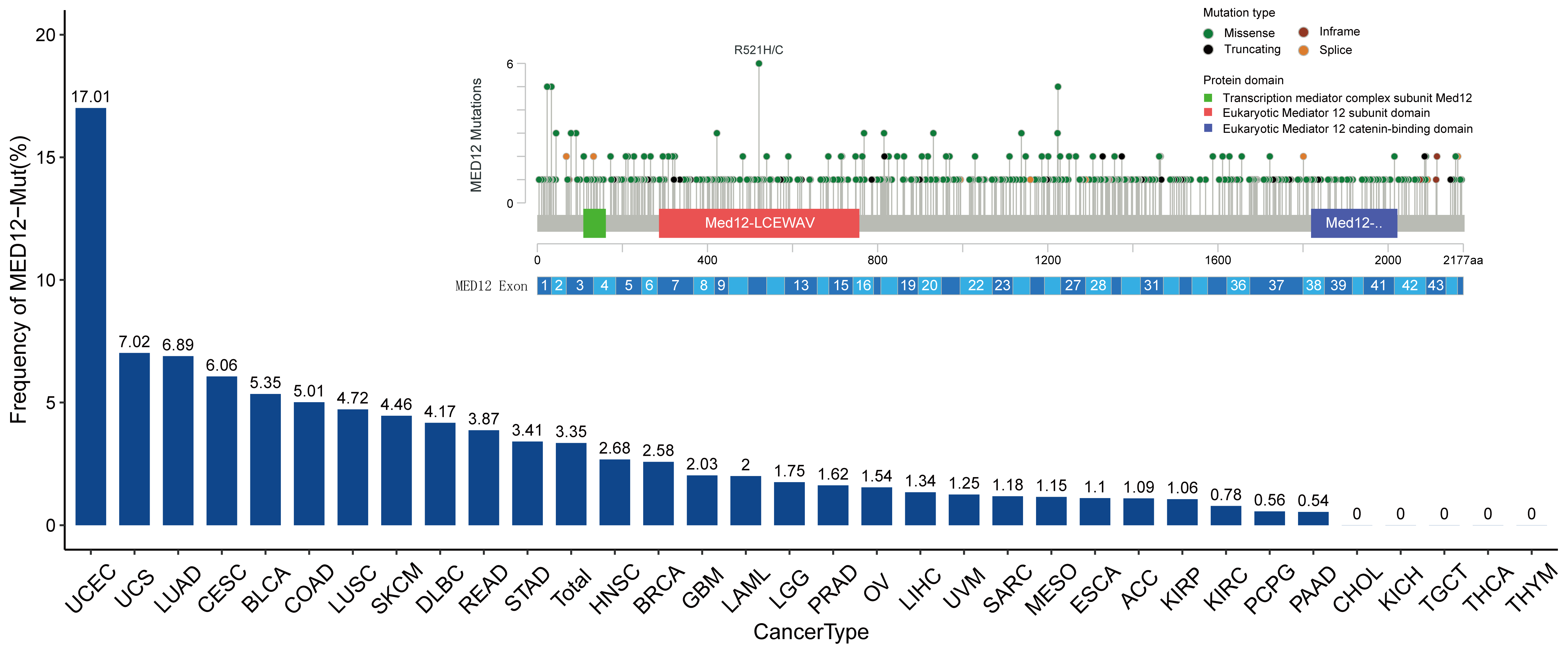

Supplement: Supplementary file 2 — Additional file 2: Fig. S2. The pan-cancer landscape of MED12 mutations across human tumors. The proportion of MED12 mutated tumors identified for each cancer type with alteration frequency in TCGA pan-cancer cohorts. [file 40001_2022_856_MOESM2_ESM.png]

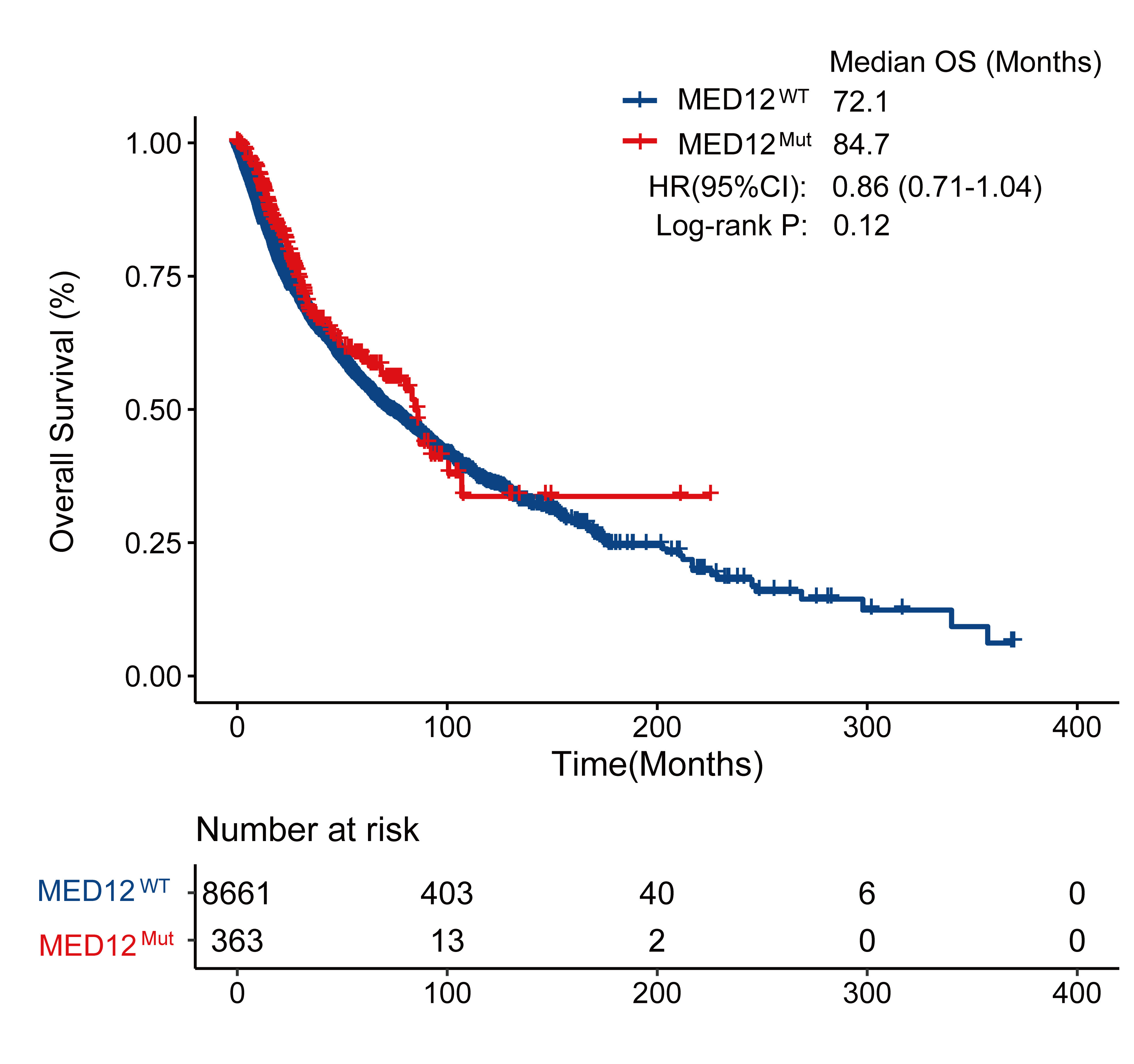

Supplement: Supplementary file 3 — Additional file 3: Fig. S3. Kaplan–Meier curves of OS between the MED12-Mut and wildtype groups in the TCGA cohort. [file 40001_2022_856_MOESM3_ESM.png]
