# Supplementary material for: MED12 mutation as a potential predictive biomarker for immune checkpoint inhibitors in pan-cancer
Source: Eur J Med Res. 2022 Oct 29;27:225. doi: 10.1186/s40001-022-00856-z (PMC9618186; doi:10.1186/s40001-022-00856-z)
Supplement: Supplementary file 4 — Additional file 4: Materials and methods [file 40001_2022_856_MOESM4_ESM.docx]

**Material and methods**

**Clinical cohorts (WES and MSKCC cohort)**

To evaluate the prediction value of MED12 gene in ICIs-treated cancers, we collected whole exome sequencing (WES) data and corresponding clinical information from Hellman, Rizvi, Miao, Allen and Liu cohort (Supplementary figure S1) across 5 cancer types consoled as WES cohort[1-6]. The mutation data were obtained from the cBioPortal website (https://www.cbioportal.org). To validate the predictive function of MED12 mutation, an independent pan-cancer cohort by Samstein et al[7]. the clinical and genomic data was also retrieved from cBioPortal (https://www.cbioportal.org). Samples from this cohort were sequenced using MSKIMPACT panels, including 341-, 410- and 468-gene panel. The Inclusion and Exclusion criteria are shown in Supplementary Figure S1. All nonsynonymous somatic mutations, including nonsense, missense, nonstop, frameshift deletion and insertion, in-frame deletion and insertion, and splice site mutations were considered for inclusion in our study. MED12-mutant and MED12-wildtype (MED12-Wt) tumors were defined as with and without nonsynonymous somatic mutations of MED12, respectively.

We only used the data from pre-treatment biopsy samples because patients' mutation status would change following ICIs treatment. In addition, duplicate samples were also excluded. Furthermore, we validated the predictive value of MED12-Mut in MSKCC cohort. Processing and analyzing of validation cohort were shown in the Supplementary Figure S1.

**Clinical outcomes**

The primary clinical outcomes were progression-free survival (PFS), overall survival (OS) and objective response rate (ORR). Tumor response, including complete response (CR), partial response (PR), or stable disease (SD), for patients was defined by RECIST or RECIST v1.1. PFS was defined as the date that the patient began ICI treatment to the date of progression or death of any cause. And patients without progression were censored at the date of their last scan. OS was defined as from the date of ICIs treatment in immunotherapy cohort, or from the date of the first non-ICIs (target-therapies/chemotherapies) treatment in the non-ICI treated cohort respectively.

**The Cancer Genome Atlas (TCGA) cohort**

Somatic mutations of 10953 patients across 33 tumor types were retrieved from the UCSC Xena data portal (https://xenabrowser.net) [8], which were used for the analysis of MED12-Mut frequency and mutation distribution. Nonsynonymous mutations in coding region of MED12 gene were defined as MED12 mutation. MutationMapper module (https://www.cbioportal.org/mutation_mapper) from the cBioPortal was used for investigate the distribution of mutations at the protein domain.

**Evaluation of prognostic value**

The survival data of patients without ICIs treatment were retrieved from TCGA cohort was used to explore the prognostic influence of MED12 (Supplementary Figure S3).

**TMB and TNB analysis**

For WES-sequenced samples, the TMB was defined as the total number of nonsynonymous mutations divided by the exome size (38 Mb). For MSK-IMPACT panel sequenced samples, the total number of nonsynonymous mutations was normalized to the exonic coverage of the MSK-IMPACT panel (1.22, 1.06, and 0.98 Mb in the 468-, 410-, and 341-gene panels, respectively). Tumor neoantigen burden (TNB) of WES cohort samples were obtained from cBioPortal website (https://www.cbioportal.org).

**Correlation analysis with DDR pathway**

DNA damage response (DDR) pathway genes (Supplementary Table S1) that acquired from the Broad Institute Molecular Signatures Database (MSigDB) [8] to compare the differences in the mean mutation number of DDR pathway between MED12-Mut and MED12-Wt in WES cohort.

**Correlation analysis for tumor immunogenicity and immune features**

The TIMER2.0 (http://timer.cistrome.org/) was used for the systematic analysis of immune infiltrates across different cancer types from TCGA include 6 Immune cell types（B cells, CD4+ T cells, CD8+ T cells, Neutrphils, Macrophages and Dendritic cells）. The TIMER immune infiltration proportions were obtained from the project implemented by Li et al[9].

**Statistical analysis**

Continuous variables were compared by Mann-Whitney U test and categorical variables were compared by Chi-square test or Fisher’s exact test. Kaplan-Meier curve (K-M curve) analysis of PFS and OS was compared by the log-rank test. The Cox proportional hazards regression was applied for univariable and multivariate analysis, and available confounding factors including sex, age, metastasis status, treatment, TMB level and cancer type were adjusted. Proportional hazards assumption was tested before the Cox regression. Then we used the Stratified Cox model to resolve independent variables that do not conform to the PH assumption. Variables with P < 0.1 in the univariable regression and those which has been reported linked with efficacy of immunotherapy were also included into multivariable Cox regression. All reported P values were two-tailed, and P < 0.05 was considered statistically significant. Statistical analyses were performed using R v. 4.0.3 (https://www.r-project.org).

References

1. M. D. Hellmann, T. Nathanson, H. Rizvi, B. C. Creelan, F. Sanchez-Vega, A. Ahuja, et al. Genomic Features of Response to Combination Immunotherapy in Patients with Advanced Non-Small-Cell Lung Cancer. Cancer Cell. 2018; 5:843-852 e4; <https://10.1016/j.ccell.2018.03.018>.

2. N. A. Rizvi, M. D. Hellmann, A. Snyder, P. Kvistborg, V. Makarov, J. J. Havel, et al. Cancer immunology. Mutational landscape determines sensitivity to PD-1 blockade in non-small cell lung cancer. Science. 2015; 6230:124-8; <https://10.1126/science.aaa1348>.

3. D. Miao, C. A. Margolis, N. I. Vokes, D. Liu, A. Taylor-Weiner, S. M. Wankowicz, et al. Genomic correlates of response to immune checkpoint blockade in microsatellite-stable solid tumors. Nat Genet. 2018; 9:1271-1281; <https://10.1038/s41588-018-0200-2>.

4. D. Miao, C. A. Margolis, W. Gao, M. H. Voss, W. Li, D. J. Martini, et al. Genomic correlates of response to immune checkpoint therapies in clear cell renal cell carcinoma. Science. 2018; 6377:801-806; <https://10.1126/science.aan5951>.

5. E. M. Van Allen, D. Miao, B. Schilling, S. A. Shukla, C. Blank, L. Zimmer, et al. Genomic correlates of response to CTLA-4 blockade in metastatic melanoma. Science. 2015; 6257:207-211;

6. D. Liu, B. Schilling, D. Liu, A. Sucker, E. Livingstone, L. Jerby-Arnon, et al. Integrative molecular and clinical modeling of clinical outcomes to PD1 blockade in patients with metastatic melanoma. 2019; 12:1916-1927;

7. R. M. Samstein, C. H. Lee, A. N. Shoushtari, M. D. Hellmann, R. Shen, Y. Y. Janjigian, et al. Tumor mutational load predicts survival after immunotherapy across multiple cancer types. Nat Genet. 2019; 2:202-206; <https://10.1038/s41588-018-0312-8>.

8. M. J. Goldman, B. Craft, M. Hastie, K. Repečka, F. McDade, A. Kamath, et al. Visualizing and interpreting cancer genomics data via the Xena platform. Nat Biotechnol. 2020; 6:675-678; <https://10.1038/s41587-020-0546-8>.

9. T. Li, J. Fu, Z. Zeng, D. Cohen, J. Li, Q. Chen, et al. TIMER2.0 for analysis of tumor-infiltrating immune cells. Nucleic Acids Res. 2020; W1:W509-w514; <https://10.1093/nar/gkaa407>.
